# Improving Physical Health Data Provided on Discharge Summaries From Sheffield Home Treatment Team

**DOI:** 10.1192/bjo.2022.321

**Published:** 2022-06-20

**Authors:** Ross Nieuwoudt, Alex Bradwell, Gaelle Slater

**Affiliations:** Sheffield Health and Social Care, Sheffield, United Kingdom

## Abstract

**Aims:**

The aim of this QIP was to identify whether the information obtained during routine physical health reviews was being adequately handed over during discharge from the Sheffield Home Treatment Team within discharge summaries. Individuals with serious mental illness have a significantly higher all-cause mortality rate than those without, much of which is due to preventable physical health conditions. Due to this, the Home Treatment Team aims to complete a Physical Health Review (PHR) for every patient under their care as per guidelines. It is important that these examinations are performed, however, it is equally important that the results of PHRs are communicated to the patient's primary care physician upon discharge. Communication between services is vital for continuity of care and to ensure that identified problems are managed effectively. On discharge, relevant data from these investigations should be communicated to the service user's registered GP surgery for appropriate follow-up care. Performing investigations without informing the service user's primary physician is an inefficient use of resources and may result in unnecessary repeated investigations and procedures. There is not currently an official system in place to assure that the investigations and results of PHRs are summarised and communicated upon discharge.

**Methods:**

There were two steps taken in this stage of the QIP. First, a questionnaire was distributed to all members of the Sheffield Home Treatment Team, including medics, nurses, and STR workers. The responses were compiled and analysed to form the criteria and standards for an audit of previous discharges. Following this, an audit was performed for the months of June-July 2021, data were kindly collected by junior doctors. This data looked to determine whether previous discharges met the criteria and standards set by the questionnaire.

**Results:**

The results of the audit showed that the discharges did not meet the standards set, with many containing little to no information. Only 49% of the service users with physical health reviews had any information provided on discharge. Of these, the contents of the summaries were varies and inconsistent, resulting in a significant amount of information becoming unavailable to the service user's GPs.

**Conclusion:**

- The current system is insufficient in terms of handing over physical health information collected during investigations performed by the Home Treatment Team.
- A proposed solution will be implemented in the coming months.
- A re-audit will be performed to complete the audit cycle and assess the efficacy of the proposed solution.

